# Novel genetic tools that enable highly pure protein production in *Trichoderma reesei*

**DOI:** 10.1038/s41598-019-41573-8

**Published:** 2019-03-22

**Authors:** Anssi Rantasalo, Marika Vitikainen, Toni Paasikallio, Jussi Jäntti, Christopher P. Landowski, Dominik Mojzita

**Affiliations:** VTT Technical Research Centre of Finland, P.O. Box 1000, FI-02044 VTT Espoo, Finland

## Abstract

*Trichoderma reesei* is an established protein production host with high natural capacity to secrete enzymes. The lack of efficient genome engineering approaches and absence of robust constitutive gene expression systems limits exploitation of this organism in some protein production applications. Here we report engineering of *T*. *reesei* for high-level production of highly enriched lipase B of *Candida antarctica* (calB) using glucose as a carbon source. Multiplexed CRISPR/Cas9 in combination with the use of our recently established synthetic expression system (SES) enabled accelerated construction of strains, which produced high amounts of highly pure calB. Using SES, calB production levels in cellulase-inducing medium were comparable to the levels obtained by using the commonly employed inducible *cbh1* promoter, where a wide spectrum of native enzymes were co-produced. Due to highly constitutive expression provided by the SES, it was possible to carry out the production in cellulase-repressing glucose medium leading to around 4 grams per liter of fully functional calB and simultaneous elimination of unwanted background enzymes.

## Introduction

The use of recombinant proteins in various industries is steadily increasing. For instance, the chemical industry aims at novel practices, where use of enzymes would ensure improved process performance, increased safety, and lower impact on the environment^1^. In addition, numerous medical applications are increasingly employing protein-based therapeutics (biologics), which provide more efficient treatments or more precise diagnostics^2,3^. However, one of the challenges limiting faster progress in these applications is the lack of efficient high-level and low cost protein production platforms.

The filamentous fungus *Trichoderma reesei* (*T*. *reesei*) is an important and widely exploited organism for production of industrial enzymes with a high, natural capacity to secrete cellulolytic enzymes^4^. This property has made it an attractive host for multiple industrial applications^5,6^, particularly for production of cellulases, xylanases, amylases, and proteases^5–8^. Recently, *T*. *rees*ei has also been proposed to provide a low-cost production platform for biopharmaceuticals, such as interferons^9,10^. Some studies have reported total secreted protein titers exceeding 100 g/L, which highlight the productivity potential of this organism for protein production applications^11^.

The most often-used protein expression systems in *T*. *reesei* are based on the use of native cellulolytic gene promoters, especially cellobiohydrolase 1 (*cbh1*) promoter^5,6,12,13^. These promoters are strongly activated in the presence of inducing compounds, mainly cellulose or its derivatives, but also lactose or other compounds^14,15^. The transcriptional regulation of cellulolytic genes is highly coordinated by transcription factors such as XYR1, CREI and ACEI^14,16–18^. Thus, exposure to inducing compounds results in simultaneous expression and secretion of a wide spectrum of cellulolytic and hemicellulolytic enzymes^15^. In inducing conditions, approximately 80–85% of secreted proteins are cellobiohydrolases, but also endoglucanases and xylanases are strongly produced^7,16^. Cellobiohydrolase I (CBHI) alone represents about 50–60% of total secreted proteins^7^. While co-expression of the cellulolytic enzymes can be a highly useful for production of cellulolytic enzyme cocktails^7^, these side activities can be undesired or harmful when heterologous proteins are produced in these conditions^19,20^.

Protein purification from the typical production culture supernatant containing a wide mixture of enzymes may require extensive downstream processing and thus significantly affects the overall economic and technical feasibility of the bioprocess, especially considering the industrial scale of a typical cultivation^19^. Partly due to these reasons, *T*. *reesei* has not been extensively used as a host in applications in which purity of the end-product is required. To minimize co-production of unwanted proteins, deletion of the major cellulase genes or the main transcriptional regulator *xyr1*^21^ can provide a partial solution. However, this may not be desired and straightforward, because the lack of cellulase activities limits conversion of carbon source substrate into smaller inducing molecules and may decrease the production output of *T*. *reesei*, particularly in cellulose-containing conditions^14^.

Several attempts have been made to develop constitutive or glucose induced/derepressed protein expression systems for *T*. *reesei*^19,20,22^. The ability to use a simple and non-inducing culture media for protein production would provide several advantages such as significant reduction of undesired background proteins, and/or improved reproducibility of the bioprocesses. This could be particularly important for processes in which good manufacturing practices (GMP) need to be followed. Unfortunately, the existing constitutive or glucose induced expression systems have not been able to provide industrially relevant yields.

To address the need for a strong, growth-condition independent expression system for *T*. *reesei*, we recently developed a universally functional synthetic gene expression system (SES) for fungal hosts^23^. The SES system is based on the synthetic transcription factor (sTF) which strongly activates the expression of downstream gene(s) controlled by an artificial sTF-dependent synthetic promoter(s). Expression of the sTF is controlled by a core promoter, instead of a full-length promoter, which results in low and highly constitutive sTF levels. The substantial benefit of this concept is minimal influence of external conditions on the expression levels of the target genes, allowing the use of media which reduces production of unwanted native proteins.

With an efficient gene expression tool at hand a challenge for *T*. *reesei* still remains for efficient and fast generation of genetically modified strains. With current approaches, genetic engineering of *T*. *reesei* is a relatively slow and work intensive process. Each strain engineering step that involves targeted genomic integration, either deleting native genes or adding heterologous genes, requires a substantial amount of time. Inefficient homologous recombination machinery, in combination with a strong preference for non-homologous recombination in *T*. *reesei*, leads to a low frequency of correctly targeted genome integration. Thus, extensive screening and strain purification is typically required to obtain strains with desired genotypes. Deletion or silencing of components of the non-homologous end-joining (NHEJ) pathway, such as the *tku70* gene, can significantly improve gene targeting efficiencies^24–26^. However, even with those modifications the inability to integrate several DNA cassettes simultaneously (multiplexing) into the desired loci has remained a bottleneck.

These challenges can be partly overcome by the use of CRISPR/Cas9 technology, which has previously been used for this purpose in various organisms, including yeast *Saccharomyces cerevisiae*, mammals, and several filamentous fungi, for instance, *T*. *reesei*^27^, *Aspergillus* species^28,29^, or *Penicillium chrysogenum*^30^. In most cases, Cas9 and a specific gRNA have been delivered into the host organisms as DNA, encoding these CRISPR components. However, constitutive or high-level production of an active Cas9 protein can lead to significantly decreased viability or genomic integrity of the host, likely due to unintended genomic modifications. Another option is direct delivery of Cas9-gRNA nucleo-protein complex into the host during transformation, together with a donor DNA as previously demonstrated in *Penicillium chrysogenum*^30^. The advantage of this approach is that there is no prior strain engineering required and cellular DNA is only temporally exposed to an active Cas9 protein. Even though this would be a favorable method, it has not been demonstrated previously in *T*. *reesei*.

In this study, we optimized the SES system and established an efficient strain engineering method based upon transient transformation of CRISPR/Cas9 protein, which facilitates multiplexed genome editing in *T*. *reesei*. The lipase B of *Candida antarctica* (calB) was used to showcase the new approaches. Lipases are an important group of enzymes, which have wide applicability in different industries, such as in food, textile, detergent, cosmetic, bioenergy and pharmaceutical industries^31,32^. Specifically, calB, has been shown to be a robust enzyme, which retains its activity in harsh industrial conditions, such as in high solvent content^33^.

## Results

### Development of an efficient CRISPR/Cas9 transformation method

We aimed to develop an efficient CRISPR/Cas9 transformation protocol for Cas9 nucleo-protein complexes to enable multiplex gene deletions/replacements in *T*. *reesei*. We tested simultaneous deletion of three major cellulase genes: cellobiohydrolase II (*cbh2*), endoglucanase I (*egl1*), and endoglucanase II (*egl2*). The deletion of these genes would lead to a strain with significantly reduced production of background proteins in the culture supernatant. In addition, the deletion of major cellulase genes was suggested to decrease congestion in the secretory pathway^34^. The cellobiohydrolase I (*cbh1*) was not selected for this test, since the *cbh1* locus is a common site for targeted integration of the heterologous gene expression cassette, which results in the *cbh1* coding region replacement by the target gene, utilizing the *cbh1* promoter for the expression in the final production strain.

**Figure 1:**
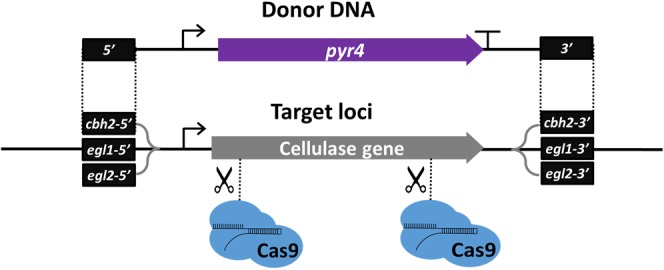
Scheme of the CRISPR/Cas9-mediated multiplexed gene deletions in *T*. *reesei*. The donor DNA molecules were constructed, each containing the *pyr4* selection marker gene flanked by ~1000 bp of DNA region homologous to 5′-upstream and 3′-downstream regions of the selected gene. Two gRNAs were designed to target Cas9 protein for introducing double stranded break at the 5′- and 3′-ends of each targeted coding sequence. A single-transformation was performed combining 6 *in vitro* pre-assembled Cas9 ribonucleo-complexes, three donor DNAs, and the *T*. *reesei* protoplasts. Simultaneous deletion of the three cellulase genes, cellobiohydrolase II (*cbh2*), endoglucanase I (*egl1*), and endoglucanase II (*egl2*), was achieved in about 12% of the resulting colonies (Table 1).

In a single transformation event, two gRNAs targeting the Cas9 complex into the 5′- and 3′- regions of each gene were used together with three donor DNA molecules, each encoding *pyr4* marker gene flanked with DNA homologous to the genomic regions outside the Cas9 target sites (Fig. 1). Two hundred transformants were streaked on selective plates, and 193 viable colonies were tested by PCR for the integration of *pyr4* marker into the correct locus and for presence of the intact target gene. Clear PCR products were obtained from 139 colonies (72% of total number of viable transformants) indicating either correct integration of the *pyr4* marker or intact coding region of *cbh2*/*egl1*/*egl2* gene (Table 1). Since the PCR screening was carried out directly from streaks without a single colony purification step, some of the isolates turned out to be mixed populations (both corresponding PCR products obtained). In 16 (12%) out of 139 colonies, all three target genes were correctly and simultaneously deleted; 11 pure clones were obtained directly, and additional 5 clones were obtained by purification of mixed colonies by plating diluted conidia and re-analyzing the resulting colonies by PCR. Altogether 24% of the colonies had two deletions, and all three combinations of double deletions were identified. The majority of the colonies (64%) were single deletions and the frequency of single deletion was rather similar with all three loci (19–23%).

**Table 1:** PCR analysis of clones obtained by the CRISPR/Cas9 protein transformation simultaneously targeting three loci (*cbh2*, *egl1*, and *egl2*).

	Triple deletion	Double deletion	Double deletion	Double deletion	Single deletion	Single deletion	Single deletion	Total
	Δ*cbh2* Δ*egl1* Δ*egl2*	Δ*cbh2* Δ*egl1*	Δ*chb2* Δ*egl2*	Δ*egl1* Δ*egl2*	Δ*cbh2*	Δ*egl1*	Δ*egl2*	
Number of clones	16 (12%)	9 (6%)	10 (7%)	15 (11%)	27 (19%)	30 (22%)	32 (23%)	139
Pure clones (% of total)	11 (69%)	8 (89%)	9 (90%)	10 (67%)	23 (85%)	28 (93%)	31 (97%)	120 (86%)
Mixed clones (% of total)	5 (31%)	1 (11%)	1 (10%)	5 (33%)	4 (15%)	2 (7%)	1 (3%)	19 (14%)

### The synthetic expression system optimized for *T*. *reesei*

The previously established universal synthetic expression system (SES), functional in a wide spectrum of fungal hosts, was based on the use of core promoters (*cp*) originating from *Aspergillus niger*^23^. The sTF, in that expression system, was constitutively expressed from either *An-008*cp or *An-533*cp. In all the tested organisms, especially in *T*. *reesei*, very high expression levels were observed for the target genes controlled by the sTF-dependent promoters. However, we observed that a mild increase of the sTF expression could still lead to improved performance of the system, while excessively high sTF expression led to toxic effects such as decreased growth rates or lethality (our unpublished observations).

**Figure 2:**
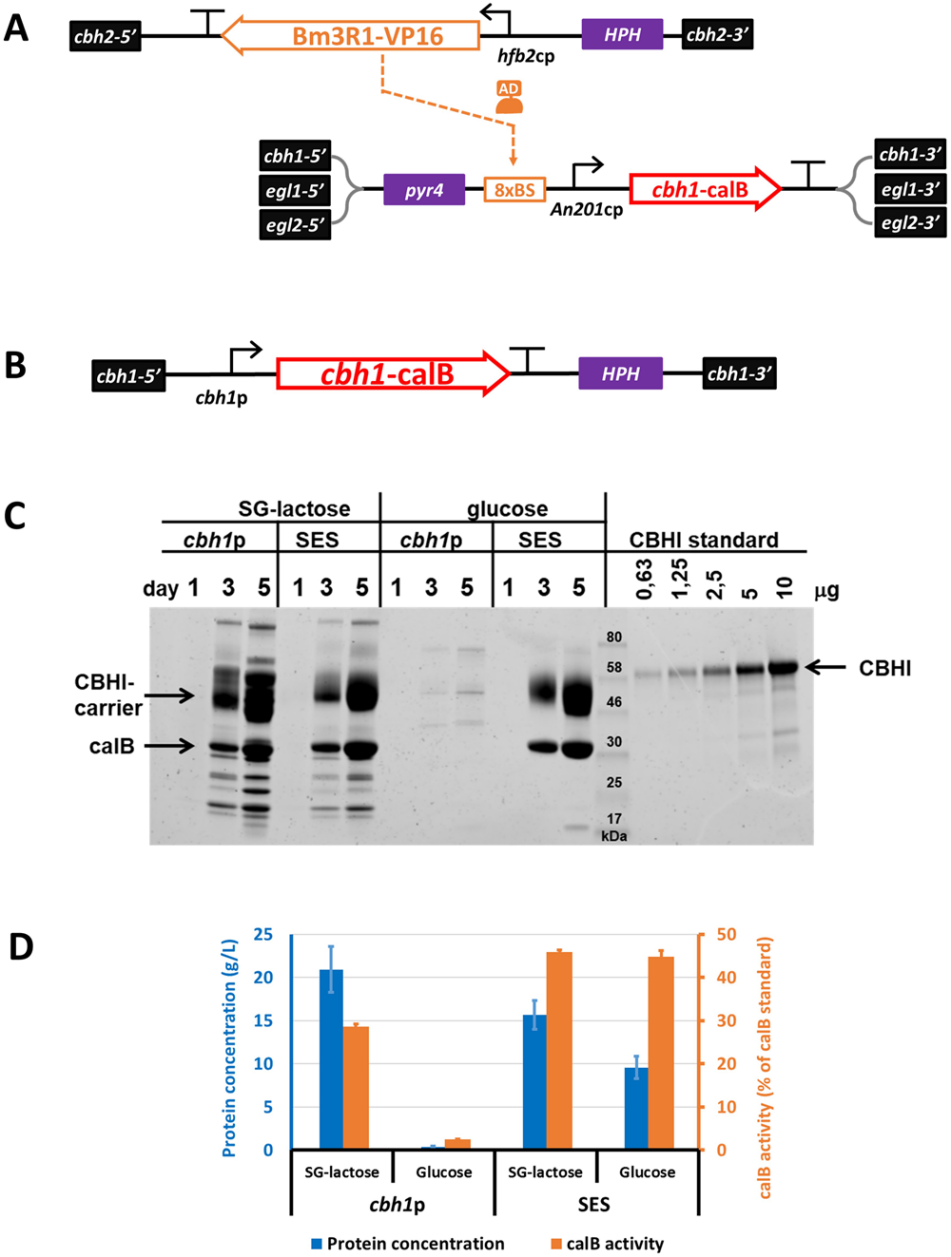
Comparison of approaches for the calB production in *T*. *reesei*. (**A**) Scheme of the synthetic expression system used for the CBHI-calB production. The expression of synthetic transcription factor (sTF; Bm3R1-VP16) was driven by the *hfb2* core promoter (*T*. *reesei* origin), which provides a low and constitutive sTF expression via its basal transcription activity. The sTF recognizes the binding sequences (8 × BS) in an engineered promoter of the *cbh*1-*calB* gene. The sTF binding sites are followed by the *An201* core promoter (*A*. *niger* origin), forming the synthetic promoter. The sTF expression cassette was integrated in a single copy into the *cbh2* locus and the CBHI-calB expression cassette was integrated as three copies in the *cbh1*, *egl1* and *egl2* loci. (**B**) Scheme of a control CBHI-calB expression cassette. The *cbh1*-*calB* gene was expressed from the commonly used cellulose inducible *cbh1* promoter. The cassette was integrated into *cbh1* locus in a single copy. (**C**) The SDS-PAGE analysis of proteins produced (secreted) into culture media of bioreactor cultivations. The strains carrying expression cassettes shown in (**A**) (SES strain) and (**B**) (*cbh1*p strain) were cultivated either in SG-lactose medium or in glucose-containing medium. The cultivation media samples were collected as indicated and diluted 1:10 in water prior to analysis. Purified, full length CBHI protein was used as a loading standard and the proteins were visualized by Coomassie staining. (**D**) The total protein concentrations (blue bars, left y-axis) and the calB lipase activities (orange bars, right y-axis) in the samples collected on day 5 of the bioreactor cultivations. The lipase activity levels were normalized to total protein concentrations and the results are presented as a percent of commercially available calB activity. The values and the error bars represent means and standard deviations from three technical replicates.

To increase expression of the sTF gene in *T*. *reesei*, we tested additional core promoters to be used for its expression in the optimized SES system. We focused on selected core promoters originating from *T*. *reesei*, previously screened in *S*. *cerevisiae*^23^. The hydrophobin II core promoter (*hfb2*cp, previously named as *Tr_119989*cp), used in the modified SES reporter system for the expression of sTF, provided the highest levels of the mCherry-reporter production (data not shown). Therefore, it was used for constructing the sTF expression cassette (Fig. 2A). To create a SES background strain, the sTF expression cassette was single-copy integrated into one of the four major cellulase gene loci, namely the *cbh2* locus, of the *T*. *reesei* genome. The other three loci - *cbh1*, *egl1*, and *egl2* - were used for genomic integration of target gene expression cassettes. The multiplication of the target gene cassette was expected to further increase the expression of the target gene.

### The SES target gene expression cassettes

The lipase B of *Candida antarctica* (calB) was selected as an example target protein to demonstrate the utility of the SES for the production of an industrially relevant product. Three expression cassettes were constructed, where the expression of *calB* was controlled by a synthetic promoter containing eight sTF binding sites positioned upstream the *An201* core promoter (Fig. 2A). The *calB* coding region was modified by replacing native N-terminal secretion signal with CBHI carrier protein and Kex2 cleavage site encoding sequences^10^. The N-terminal part of CBHI is commonly used as a fusion carrier protein for facilitating heterologous protein secretion into culture medium^10,35–37^. The high natural capacity of *T*. *reesei* to secrete CBHI protein is utilized in these applications to provide a strong N-terminal secretion signal sequence in a context of the CBHI protein, which often leads to improved protein product secretion, folding, and stability. As a comparative example for the SES-based production, a classical approach was tested where an identical CBHI-carrier-calB fusion gene was placed under control of the *cbh1* promoter (Fig. 2B).

The SES-calB expression cassettes were used as donor DNA molecules, in CRISPR/Cas9-transformation protocol developed above, for simultaneous deletions/replacements of *cbh1*, *egl1*, and *egl2* genes in the *T*. *reesei* strain carrying the sTF expression cassette. This resulted in a SES strain with four major cellulase genes replaced by the SES-based calB production platform (Fig. 2A). In parallel, the calB cassette with the *cbh1* promoter was integrated as a single copy into *cbh1* locus to create a control strain (Fig. 2B).

### The calB lipase production

The calB production was tested in fed-batch bioreactor cultivations. The strains were grown using commonly used cellulase inducing medium (containing spent grain and lactose; SG-lactose) and cellulase repressing medium (containing glucose). In the inducing medium, both strains showed similar calB production during the fermentation (Fig. 2C). In addition, based on the SDS-PAGE analysis, the calB protein (33 kDa) together with the CBHI carrier (56 kDa) were the most abundant products (Fig. 2C). The total secreted protein concentration in the end of fermentation (day 5) was 20.9 g/L in control strain (*cbh1*p) and 15.7 g/L in SES strain (Fig. 2D). As expected, the overall production of native proteins was substantially decreased in the SES strain (Fig. 2C).

Once the cultivation was performed in the glucose-containing medium, dramatic reduction in protein production was observed in the control strain (Fig. 2C), since the total secreted protein concentration decreased to 0.4 g/L which corresponds to a ~98% reduction in extracellular protein concentration (Fig. 2D). In contrast to this, the production of calB was virtually unaffected in the SES-strain, where the calB and the CBHI-carrier protein seemingly constitute the only secreted products. Reduction in total protein concentration decreased from 15.7 g/L to 9.6 g/L (~39% reduction), which can be accounted for repression of native cellulolytic genes in the presence of glucose. This demonstrated a superior stability of expression provided by the SES system.

To assess the activity of the produced calB, we performed an enzymatic lipase assay from samples collected at day 5 of the bioreactor cultivations. The lipase activity was normalized to total protein concentrations in each sample and the activities were expressed as percentage of the commercially available calB enzyme activities (Fig. 2D). The calB activities varied from 2.6% to ~45% of the commercial enzyme. Interestingly, the calB produced by the SES-strain showed similar levels of normalized activity in both cultivation conditions (~45% of commercial calB in SG-lactose as well as in glucose medium), even though the SDS-PAGE indicated higher purity of the product derived from glucose cultivation. The overall protein yields were satisfactory in terms of achieved product concentration; however, the presence of the CBHI protein carrier compromised the product purity. To accomplish the calB purity at similar or higher levels than in the commercial calB preparations, we attempted to replace the CBHI-carrier protein with a short secretion signal peptide.

### Use of a short secretion signal peptide

**Figure 3:**
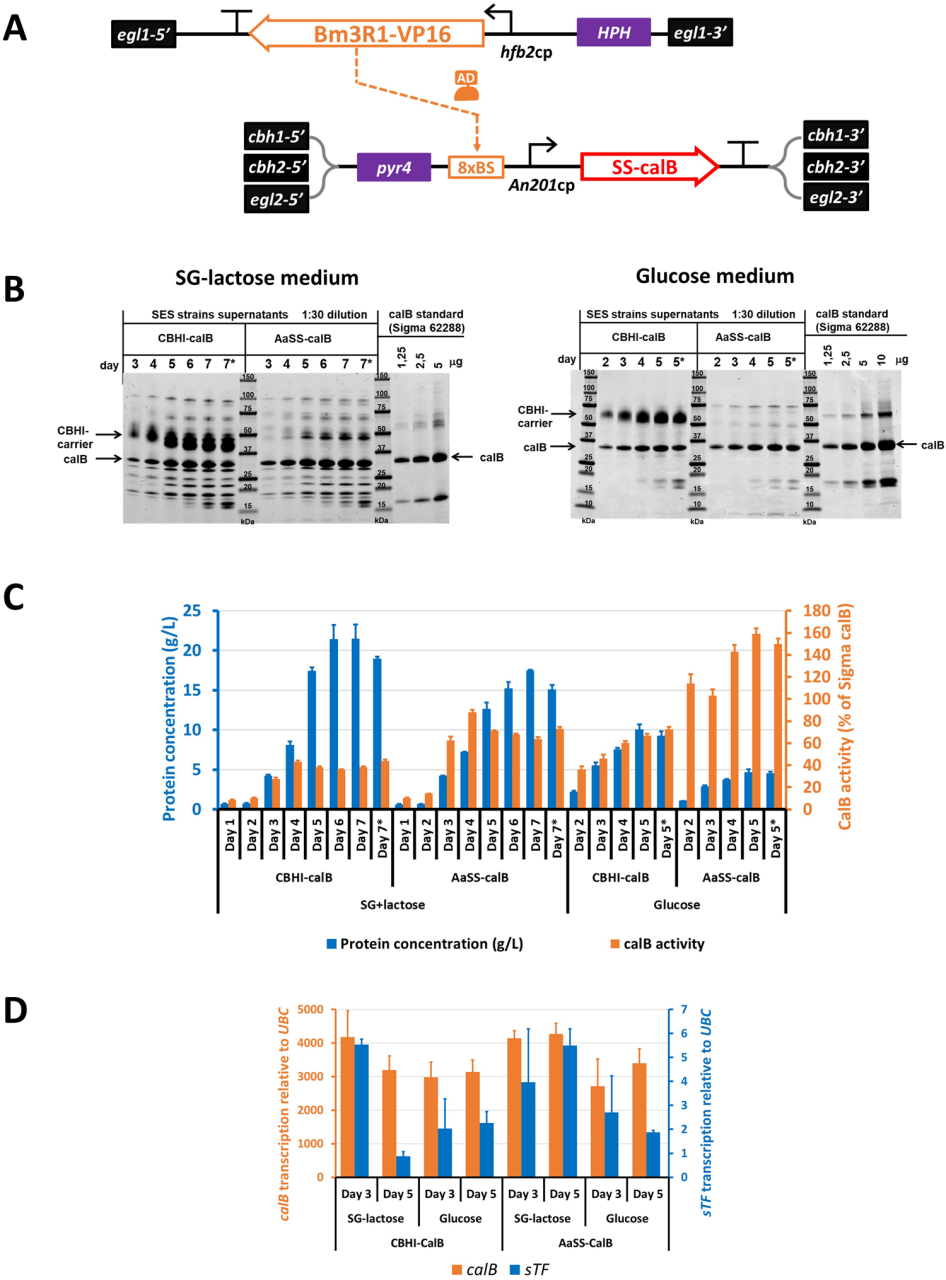
Production of highly enriched calB protein using a short secretion signal sequence. (**A**) Scheme of the SES system used for the production of calB with a short secretion signal sequence (SS; 26 N-terminal amino-acids from *A*. *awamori* α-amylase). The sTF expression cassette was integrated into the *egl1* locus (single copy) and the AaSS-calB expression cassette was integrated into the *cbh1*, *cbh2* and *egl2* loci (triple copy). (**B**) The SDS-PAGE analysis of proteins produced (secreted) into culture media of bioreactor cultivations. The strain producing CBHI-calB (SES strain in Fig. 2) and the AaSS-calB strain carrying the expression cassettes from (**A**) were cultivated either in SG-lactose medium for 7 days or in glucose-containing medium for 5 days. The cultivation media samples were collected as indicated and diluted 1:30 in water prior to analysis. Commercially available calB protein was used as a loading standard and the proteins were visualized by Coomassie staining. The samples from the last day of each cultivation subjected to gel-filtration chromatography and freeze-drying were also analyzed after reconstitution in water and 1:30 dilution (marked with asterisk). (**C**) The total protein concentrations (blue bars, left y-axis) and the calB lipase activities (orange bars, right y-axis) in the samples collected from the bioreactor cultivations and in the gel-filtrated, freeze-dried samples (marked with asterisk). The lipase activity levels were normalized to total protein concentrations and the results are presented as a percent of commercially available calB activity. The values and the error bars represent means and standard deviations from three technical replicates. (**D**) Transcription analysis of strains cultivated in bioreactors. The calB and sTF transcript levels were normalized to the transcript levels of native *ubc* gene. Transcript levels of the sTF are shown on the secondary (right) y-axis. The values and the error bars represent means and standard deviations from two biological (four technical) replicates.

To eliminate use of the CBHI-carrier protein, we looked for secretion signal peptides which, when fused to a protein of interest, would enable efficient secretion. We selected four previously identified secretion signal peptides of well-secreted fungal enzymes: CBHI from *T*. *reesei*^38^, CBHII from *T*. *reesei*^39^, α-amylase from *Aspergillus awamori*^39^ and glucoamylase from *Aspergillus niger*^39^ (Supplementary Table 1). The selected secretion signals were cloned into calB expression cassettes to form the N-terminus of calB protein (SS-calB). The *calB* expression cassettes had otherwise identical structure to those used for the expression of calB with the CBHI-carrier protein; however they contained a different set of targeting regions for the genome integrations, i.e. parts homologous to 5′- and 3′- regions of *cbh1*, *cbh2*, and *egl2* genes (Fig. 3A). The sTF expression cassette, identical to the one used for CBHI-carrier-calB production strain, was integrated into the *egl1* locus of the *T*. *reesei* genome replacing the native gene region. The *egl1* locus was selected, instead of the previously used *cbh2* locus, based on the results indicating that expression of sTF from the *egl1* locus could provide a more stable expression pattern compared to the *cbh2* locus (Fig. 3D).

The *T*. *reesei* strain carrying a single-copy integrated sTF-cassette in the *egl1* locus was transformed by four sets of SS-calB expression cassettes in four CRISPR/Cas9-multiplex-transformations, aiming for simultaneous deletions/replacements of *cbh1*, *cbh2*, and *egl2* genes. To quickly assess the effect of the signal sequences on the production of calB, several randomly picked colonies from each transformation were cultivated in glucose-containing medium and the culture supernatants analyzed by SDS-PAGE (Supplementary Fig. 1). In addition, the calB activity was measured in a subset of the culture supernatants (Supplementary Fig. 2). Based on these initial analyses, α-amylase and glucoamylase secretion signals provided substantially more efficient secretion of calB as compared to CBHI and CBHII signal sequences. Clones with the highest calB yields, including one clone producing calB with the CBHII-SS, were analyzed for the correct genomic integrations into *cbh1*, *cbh2*, and *egl2* loci, and for the correct copy-number of the calB expression cassettes. All except one of the high-calB-producing clones showed incorrect genetic patterns, mostly multiple extra integrations events of the calB-cassettes (data not shown). The only correct clone was the calB-producer with the *A*. *awamori* α-amylase-SS (AaSS-calB), which was selected for subsequent analysis. To demonstrate the efficiency of the multiplexed CRISPR/Cas9 approach, the construction of the AaSS-calB strain was repeated in a separate transformation experiment, where additional correct clones were obtained (Supplementary Fig. 3).

### Production of highly pure calB

The production of calB by the SES system, in the α-amylase-SS-calB strain (AaSS-calB) and the previously constructed CBHI-carrier-calB strain (CBHI-calB), was assessed in fed-batch bioreactor cultivations. Both strains were grown in parallel either in SG-lactose medium in 1 L bioreactors for 7 days, or in glucose-containing medium in 15 L bioreactors for 5 days (Fig. 3B). The protein levels gradually increased throughout the cultivation and reached the maximum values on the last fermentation days (day 7 in SG-lactose/day 5 in glucose) (Fig. 3B,C). The total protein levels reached 21.4 g/L (SG-lactose medium) and 10.0 g/L (glucose medium) in case of the CBHI-calB strain, and 17.4 g/L (SG-lactose medium) and 4.6 g/L (glucose medium) in case of the AaSS-calB strain (Fig. 3C). Based on the SDS-PAGE, both strains produced similar amounts of calB (33 kDa) in both media; however, in the AaSS-calB cultivations the calB was undoubtedly the most abundant protein. In fact, once the AaSS-calB strain was grown in glucose medium, the purity of the calB in culture supernatant seemed considerably higher than the purity of commercially available calB (Fig. 3B).

The analysis of lipase activity in the culture supernatants confirmed that the calB produced in *T*. *reesei* was a fully functional enzyme (Fig. 3C). In particular, the normalized calB activity achieved with the AaSS-calB strain in glucose cultivation reached 159% of commercial calB enzyme activity, which was likely due to superior purity of our product. Other strains and cultivation conditions showed substantially lower normalized calB activities in the culture supernatants, which was caused by co-production of other proteins, such as CBHI-carrier or diverse native enzymes, into the culture medium. Interestingly, in the SES-strains (CBHI-calB and AaSS-calB) cultivated in SG-lactose, the specific calB activities reached the maximum values already at day 4 of the cultivations, declining in the following days (Supplementary Table 5). This might indicate faster accumulation of calB, which is expressed by the constitutively active SES system, as opposed to slower accumulation of the native enzymes, whose expression is gradually activated during the exposure to the inducing agents in the SG-lactose medium.

To further demonstrate the expression stability of the SES system, transcription analysis for the calB and the sTF genes was performed on selected samples collected from the bioreactor cultivations. The transcription was normalized to the previously used *ubc* gene^23^, encoding a homolog of *S*. *cerevisiae* ubiquitin-protein ligase (*UBC6*), which shows highly constitutive expression pattern in *T*. *reesei*^40^. Very high and similar transcription levels of the calB gene were observed in all tested conditions (Fig. 3D), which indicates very robust performance for the SES system. As expected, the transcription of the sTF-encoding gene was substantially lower than the calB (values for the sTF shown on the right y-axis in the Fig. 3D). In the glucose cultivations, the expression levels of the calB and sTF genes seemed to be somewhat lower, which might be caused by minor differences in the *ubc* expression levels in the two tested conditions. However, in case of the CBHI-calB strain, in SG-lactose medium, a more pronounced drop in the transcription was observed for the sTF in the fifth day of cultivation, which correlated with the lower expression of calB in this time point.

### Transcription analysis of secretion-related genes

Previously it was observed that the expression of genes involved in protein folding and secretion closely follows the expression pattern of the cellulolytic genes, being repressed in presence of glucose and induced in lactose^41^. In this study, we obtained similar calB production/secretion levels in cellulase-inducing (SG-lactose) as well as in repressing (glucose) medium (Figs 2C,D and 3B,C), which indicated that the amount of ER-targeted protein, rather than the carbon source, could determine the activity of the secretion machinery. To examine this phenomenon closer, we performed transcription analysis of four *T*. *reesei* genes involved in diverse phases of secretion process^42^: *sec61* encoding an ER-membrane channel involved in uptake of nascent polypeptides into the ER; *pdi1* encoding a protein disulphide isomerase; *bip1* encoding an ER-localized HSP70-family chaperon; and *nsf1* encoding a protein factor involved in multiple vesicle fusion steps. In the SES-based calB-producing strains, transcription of the *sec61*, *bip1*, and *nsf1* genes showed similar patterns in both cultivation conditions (Supplementary Fig. 4). The expression of the *pdi1* gene was, however, higher in SG-lactose medium compared to glucose medium. In addition, its expression also showed an increasing trend between the day 3 and 5 in the SG-lactose cultivations. In spite of some differences in the expression patterns of the secretion-related genes in the two tested conditions, the overall regulation of the secretion machinery seemed not to be significantly affected by the carbon source.

## Discussion

Development of industrial protein production strains of *T*. *reesei* often requires several simultaneous genetic modifications. These include introducing the protein product-encoding expression cassette (often in multiple copies) and deletion of selected native genes, such as proteases or cellulases. These actions are taken to maximize the productivity during extended cultivations, to decrease proteolytic activity, or to avoid secretion of unwanted proteins. However, due to inefficient homologous recombination in *T*. *reesei*, performing the targeted genetic changes is a tedious and time consuming process.

The use of CRISPR/Cas9 has been successfully established in *T*. *reesei*^27^, where up to three simultaneous genes deletions were demonstrated in a strain carrying a Cas9 expression cassette integrated in the genome. To eliminate the need for prior strain engineering, we employed the Cas9 protein and *in vitro* gRNA transformation protocol^30^ and showed feasibility of the approach for simultaneous triple deletions with a 12% frequency (Table 1). This, to our knowledge, is the highest frequency for triple deletion reported with CRISPR/Cas9 in *T*. *reesei*. In contrast to the one previous report, we achieved this by transient introduction of Cas9 and gRNA complex without a prior strain engineering step. This also dramatically reduces the probability for unwanted off target effects that may occur with a constitutively expression of Cas9. By multiplexing genetic modifications, a substantial amount of working time can be saved in strain engineering, compared to traditional transformation methods, where only single changes can be introduced at one time.

Downstream processing and protein purification are among the most cost intensive phases of protein production processes. To make the process cost effective the protein yields should be maximized and by-product formation should be minimized in the culture supernatant. Traditional *T*. *reesei* protein production strategies, based on inducible promoter expression systems, represent a challenge in this regard because a spectrum of cellulolytic enzymes are produced during growth in inducing media. An appealing alternative would be efficient production in conditions where production of the majority of native secreted proteins is repressed. Previously, Li *et al*. reported production of highly enriched xylanase II in glucose medium, reaching 1.6 g/L using pyruvate decarboxylase (*pdc)* promoter^22^. Another study has reported 50–100 mg/L production of CBHI and EGLI enzymes in glucose-containing medium, by the use of an unidentified cDNA1 promoter^19^. Even though these studies represent a valuable proof-of-concept in demonstrating highly improved protein purity in the culture supernatants, the product yields are likely not sufficient for industrial use.

In this study, we took advantage of our recently established synthetic expression system (SES), which provides strong and highly constitutive expression of a target gene^23^, and we combined the CRISPR/Cas9-based deletion strategy with the SES-based production of lipase B from *Candida antarctica* (calB). The resulting strain, containing deletions of the four major cellulase genes (*cbh1*, *cbh2*, *egl1* and *egl2*) replaced by four SES-calB expression cassettes (Fig. 2A), was constructed in only two consecutive transformation rounds, with minimal time requirements for obtaining the correct clones. In a direct comparison with a control strain, where the *cbh1* promoter was used for expression of *calB*, we demonstrated similar calB production capacities in both strains in cellulase-inducing (SG-lactose) conditions (Fig. 2C). However, the real benefit of the SES-based production was clearly demonstrated in the glucose medium, where stringent repression of the native enzymes production occurs; resulting in negligible calB amounts produced by the control strain and highly enriched calB amounts in the SES-strain. With this novel approach, close to 10 g/L of total protein (mainly consisting of CBHI-carrier and calB protein) was achieved (Fig. 2C,D), which is, to the best of our knowledge, the highest reported protein production yield in *T*. *reesei* in glucose-containing medium.

To further improve the purity of the final protein product, we eliminated the use of the bulky CBHI-carrier by employing only a short secretion-signal sequence instead (Fig. 3). We tested four signal sequence/peptide candidates and found that the *T*. *reesei* native CBHI and CBHII signal peptides, when N-terminally fused to the calB protein, did not render properly functional products (Supplementary Fig. 1). The expression of these constructs resulted in a strong phenotype observed on the transformation plates as substantially smaller number of colonies and visibly slower growth, as compared to other two constructs containing heterologous signal sequences (data not shown). We did not investigate the underlying mechanism of this phenomenon. The signal sequence originating from *Aspergillus awamori* α-amylase proved to be a well-functioning option, providing efficient secretion of calB into the culture medium (Fig. 3B). The resulting (AaSS-calB) strain, carrying the same set of cellulolytic gene deletions as the CBHI-calB strain, also produced similar amounts of the calB in both tested conditions. However, the lack of the CBHI-carrier protein resulted in substantially higher purity, particularly in the glucose medium, where the calB protein constituted approximately 75% of all produced proteins as estimated by densitometry analysis of the SDS-PAGE gels shown in Fig. 3B. In addition, only few native proteins in relatively low quantities were detected in presence of glucose. Therefore, identification and deletion of the genes encoding these background proteins, might still lead to yet improved purity of the product.

The calB produced in *T*. *reesei* retained its enzymatic activity, resulting in a protein preparation with a specific activity superior to a commercial preparation (Fig. 3C and Supplementary Table 5). This indicates that the developed process can indeed provide an enzyme in relevant concentration, fulfilling high quality requirements for industry, without a need for downstream processing (purification). Components of the media could, however, interfere with some applications of the produced enzyme and the liquid formulation is not preferred for extended storage and shipping. Therefore, we performed gel-filtration chromatography followed by freeze-drying on selected calB samples. These procedures did not have significant impact on the calB activity, as determined by the enzymatic assays of the reconstituted samples (samples marked with asterisk, Fig. 3C and Supplementary Table 5), which indicates a viable strategy for the storage and delivery of calB products.

The highly constitutive SES system could provide a number of advantages for the protein production, for instance, unlike the inducible expression systems, it could provide faster production dynamics resulting in shorter time needed for the production bioprocess. In SG-lactose conditions, we observed faster onset of calB production compared to native proteins in both SES strains, as indicated by the profile of calB activity normalized to the total accumulated proteins in the media (Fig. 3C). Nevertheless, the main benefit of the SES system is the stable performance in different conditions, especially in media eliminating co-production of the native proteins. The design of the expression system is, however, not the only feature determining the behavior of the system in the final production host. The position of expression cassettes integrated into the genome, i.e. genomic context, could also have a substantial impact on the functionality. The transcription analysis of the CBHI-calB and AaSS-calB strains in diverse conditions and phases of the cultivations (Fig. 3D) revealed such possible phenomenon. The overall transcription profiles of the sTF and calB encoding genes showed a stable pattern in both tested strains, time-points, and conditions. However, the significant sTF transcription decline observed in the CBHI-calB strain on day 5 of the SG-lactose cultivation may have been caused by the genomic locus used for integration of the sTF expression cassette. The sTF expression cassettes had the same structure in the CBHI-calB strain and in the AaSS-calB strain (Figs 2A and 3A). However, the sTF cassette was integrated into the *cbh2* locus in the CBHI-calB strain, while the *egl1* locus was used in the AaSS-calB strain. This indicates that the choice of genomic locus for integration of the gene expression cassettes plays an important role in the strain engineering efforts. In some cases it was observed that a high expression level of the sTF may have adverse effects on strain growth. Thus, when applying the SES system in *T*. *reesei* (or other hosts), a point of consideration to achieve optimal strain performance is to keep expression of the sTF at a level that does not affect overall fitness.

In conclusion, using *T*. *reesei* strain construction and production of calB lipase as a showcase, we addressed and improved several steps in the strain engineering work-flow and provided solutions for some key challenges typically associated with this process. In addition, using the SES expression system with glucose based medium, we demonstrated that *T*. *reesei* can be a suitable host for high-level production of nearly pure proteins.

## Materials and Methods

### Strains and media

The base *T*. *reesei* strain (mutagenized version of QM9414) and methods used are described in previous reports^9,23,43^. The strain contains a *pyr4* gene mutation and the *mus53* ligase gene deletion to improve homologous recombination needed for more efficient strain construction and *pyr4* marker recycling. *T*. *reesei* conidia were generated on potato dextrose agar plates (PD-A; 39 g/L potato dextrose agar).

*T*. *reesei* cultivations were conducted either in glucose-containing medium or in cellulase-inducing spent grain lactose medium (SG-lactose). The SG-lactose medium used in bioreactor cultivations contained 20 g/L spent grain extract (SGE), 20 g/L spent grain (SG), 60 g/L lactose, 36.7 mM KH_2_PO_4_, 37.8 mM (NH_4_)_2_SO_4_, 2.4 mM MgSO_4_ and 4.1 mM CaCI_2_ (pH 4.8). The glucose medium used in bioreactor cultivations contained 10 g/L glucose, 20 g/L yeast extract, 36.7 mM KH_2_PO_4_, 37.8 mM (NH_4_)_2_SO_4_, 2.4 mM MgSO_4_ and 4.1 mM CaCI_2_ (pH 4.8).

The corresponding media used in small scale cultivations (24-well plate cultivations and in bioreactor pre-cultivations) had a modified composition; The inducing medium contained 20 g/L SGE, 40 g/L lactose, 37.8 mM (NH_4_)_2_SO_4_, 36.7 mM KH_2_PO_4_, 2.4 mM MgSO_4_, 4.1 mM CaCI_2_, 15.6 µM CoCI_2_, 18.0 µM FeSO_4_, 4.9 µM ZnSO_4_ and 9.5 µM MnSO_4_; (pH 4.5). The composition of glucose-containing medium was 20 g/L glucose, 10 g/L yeast extract, 37.8 mM (NH_4_)_2_SO_4_, 36.7 mM KH_2_PO_4_, 2.4 mM MgSO_4_, 4.1 mM CaCI_2_, 15.6 µM CoCI_2_, 18.0 µM FeSO_4_, 4.9 µM ZnSO_4_ and 9.5 µM MnSO_4_ (pH 4.8). The pre-cultivation media were also supplemented with 100 µg/mL ampicillin.

### Cloning

All the plasmids used in this study were constructed using standard cloning techniques according to the manufacturer’s protocol using Gibson assembly (New England Biolabs), restriction enzyme-based techniques (Thermo Fisher Scientific), or by utilizing yeast homologous recombination^44,45^. The 5′ and 3′ flanking regions (~1000 bp each) used for the construction of cellulase deletion cassettes were amplified from genomic DNA of *T*. *reesei* by PCR using primers shown in Supplementary Table 2. The *calB* coding region used in all calB-expression cassettes contained a DNA sequence encoding amino acids 24 to 342 of the *Candida antarctica* (*Moesziomyces antarcticus*) calB protein (GenBank: CAA83122.1). The *calB* coding region was codon-optimized for *T*. *reesei*. All the PCR reactions were conducted using Kapa Hifi enzyme (Kapa Biosystems). Ligation and Gibson assembly mixes were transformed into *E*. *coli* TOP10 by electroporation and the correct plasmids were identified by analytical digestions and sequencing (Microsynth Seqlab).

### Transformations

The *cbh2*, *egl1* and *egl2* deletion cassettes, and the SES-expression cassettes targeting the *cbh1*, *cbh2*, *egl1* and *egl2* loci, were used as donor DNA molecules in the CRISPR/Cas9 transformations. These DNA-cassettes were released from the vector backbone by the *Mss*I digestion, purified, and approximately 2–4 µg of the donor DNA was used in each transformations. *T*. *reesei* transformations were conducted using the protoplast method described previously^23^. Briefly, two crRNA for each genomic target were designed and obtained from IDT. Prior to the transformations, the gRNA molecules were assembled from the crRNAs and tracrRNAs in ratio 1:1 (1 µM), and complexed with the Cas9 protein (all IDT) in equimolar ratios according to the IDT instructions. The mixture of the Cas9 nucleoproteins corresponded to the intended set of target genome modifications in each transformation reaction. The protoplasts were combined with the donor DNAs and corresponding Cas9 complexes in a transformation reaction. The selection for pyr4+ transformants were done by cultivation in media lacking uracil, and the selection of HPH+ transformants were done by cultivation in presence of 100 mg/L of hygromycin B (EMD Millipore), as described previously^23,46^.

### Genotype analysis

Colonies obtained from the transformations were re-streaked on new selection plates. Genomic DNA was extracted from the clones using Phire Plant Direct PCR Kit (Thermo Fisher Scientific). A small piece of mycelium was incubated in dilution buffer for ~two hours at 21 °C. The resulting genomic DNA sample was diluted into water, mycelium was removed by centrifugation, and the supernatant was used as a template in subsequent PCR or RT-PCR reactions.

The genotypes of calB production strains were analyzed using RT-PCR. The copy number of *calB* and sTF genes were measured by performing relative comparison to native *act1* and *xyn1* genes. The clones, which contained a single copy of the sTF expression cassette and three copies of calB expression cassette, were selected for further use. In addition, the same strategy was followed to confirm that the targeted cellulase genes had been successfully deleted. If the RT-PCR signal of the targeted cellulase gene was absent, it was considered as successfully deleted. In the case of the *cbh1*p-calB control strain (*calB* expressed from *cbh1* promoter), the strain with a single calB expression cassette in the correct locus was selected to be used in subsequent experiments. Primers used in the genotype analysis are shown in the Supplementary Table 3.

### Bioreactor cultivations

The first bioreactor cultivation set, where the production of calB was assessed in the control strain (*calB* under the control of *cbh1* promoter) and the SES-based CBHI-calB strain (Fig. 2C,D), was performed as fed-batch cultures in 1 L bioreactors (Sartorius Biostat Q Plus). Prior to inoculation, the cells for the SG-lactose fermentations were pre-cultivated in 150 mL of inducing-pre-cultivation medium (described in the “Media” section) in Erlenmeyer flasks for ~2.5 days (28 °C, 220 rpm). For the glucose cultivations, cells were pre-cultivated in 200 mL of glucose-pre-cultivation medium for ~1.5 days (28 °C, 220 rpm). One hundred mL of the pre-cultures were combined with 900 mL of either SG-lactose or glucose media in the bioreactors. Bioreactor cultivations were started in batch mode (28 °C, pH control at 4.8), and the substrate (lactose or glucose) feed was started once the initial substrates were utilized, the cultivation parameters were controlled via the control paradigm DELTABAS, as described previously^47^. Cultivation was continued for 5 days. Cultivation media samples for subsequent analysis were collected on days 1, 3 and 5.

The second bioreactor cultivation set was done using the *calB* expressing SES strains (CBHI-calB and AaSS-calB) to compare the functionality of a short signal sequence and the CBHI-secretion-carrier in the SG-lactose medium fed-batch cultures in 1 L bioreactors (Fig. 3B–D). The strains were grown in Erlenmeyer flasks for ~3 days in 200 mL of inducing-pre-cultivation medium (28 °C, 220 rpm). One hundred fifty mL of the pre-cultures were combined with 900 mL of SG-lactose medium in the bioreactors, and the cultivations were carried out for 7 days, under a regime analogous to the above described example. Cultivation was conducted in the same way as the first set was described above. The cultivations were continued for 7 day and the cultivation media samples for subsequent analysis were collected on days 1 to 7. In addition, mycelium samples for the transcription analysis were collected on days 3 and 5.

The third bioreactor cultivation set was done to compare CBHI-calB and AaSS-calB strains in the glucose containing medium in fed-batch 15 L bioreactors cultures. Two-step pre-cultures were prepared; first, the strains were grown in 200 mL of glucose-pre-cultivation medium for ~2 days (28 °C, 220 rpm) and second, the cultures were scaled-up by transfer into 1 L of fresh glucose-pre-cultivation medium and incubated for additional 1 day (28 °C, 220 rpm). The resulting cultures were used to inoculate glucose medium in 15 L bioreactors. The cultivations were conducted for 5 days following the same principles as described above, the cultivation media samples were collected on days 2 to 5, and mycelium samples for the transcription analysis were collected on days 3 and 5.

### SDS-PAGE analysis

The culture supernatant (media) samples collected during the bioreactor cultivations were diluted into water prior to loading into precast 4–20% Criterion SDS-PAGE gels (BioRad). Serial dilutions of the loading standards were prepared either from commercial calB (Sigma) or from house made purified CBHI protein solution. Seventy-five µL of the diluted samples were mixed with 25 µL of 4× loading buffer (20% glycerol, 4% SDS, 0.3 mM bromophenol blue (Merck), 10% β-mercaptoethanol, 0.1 M Tris, pH 6.8), and incubated at 95 °C for 5 minutes. Samples were cooled on ice, centrifuged, and 15 µL loaded into 4–20% Criterion SDS-PAGE gels (BioRad). After the run, gels were washed with water and stained with PageBlue Protein Staining Solution (Thermo Fisher Scientific) for 1–2 hours. Once stained, the gels were washed with water for approximately one day and the visualization of the gels were performed using the Odyssey CLx Imaging System instrument (LI-COR Biosciences).

### RNA extraction and cDNA synthesis

The mycelium samples collected during bioreactor cultivations were filtered and washed with cold water prior to freezing in liquid nitrogen. Samples were stored at −80 °C before extraction of RNA. Total RNA was extracted using the RNeasy Mini Kit (Qiagen). DNAse treatment (DNase I RNase-free, Thermo Fisher Scientific) was performed to remove residual genomic DNA from the RNA samples. Transcription First Strand cDNA Synthesis Kit (Roche) was used for the cDNA synthesis according to manufacturer’s instructions.

### RT-PCR

RT-PCR was conducted to perform genotype and transcription analysis. Diluted genomic DNA or cDNA samples were mixed with primers and LightCycler^®^ 480 SYBR Green I Master (Roche) according to manufacturer’s instructions. The RT-PCR was performed using the Lightcycler 480II device (Roche). Two technical replicates were run from each DNA target. Results were analysed using Advanced Relative Quantification Tool (Roche).

### Transcription analysis

Transcription analysis was performed on SES-CBHI-calB and SES-AaSS-calB strains **(**Fig. 3D and Supplementary Fig. 4). Samples were collected from cultivations on days 3 and 5. Two biological replicates from each strain and time point were processed and analysed as described in the sections “RNA extraction and cDNA synthesis” and “RT-PCR”. The primers which were used in transcription analysis are shown in Supplementary Table 4.

### calB activity test

The proper functionality of the produced calB enzyme was confirmed in a lipase assay. The culture supernatants collected from fermenter were diluted in water, and 20 µl of diluted samples were mixed with 180 µL of solution containing 0.25 mM p-nitro phenyl butyrate (pNPB, Sigma) dissolved in 50 mM potassium phosphate (pH 7.5) in a transparent microtiter plate (Nunc 96 F, Thermo Fisher Scientific). The formation of p-nitrophenolate (pNP), a product of enzymatic hydrolysis of pNPB, was monitored by the change in absorbance at 405 nm using Varioskan instrument (Thermo Electron Corporation). The activity was calculated from time points in which the change in absorbance was constant over time (linear relation between absorbance and time). The raw activity values were normalized to total protein concentrations in the initial samples. These results were compared to the normalized activity obtained with the commercially available calB enzyme (Sigma). The normalized activities values were divided and the calB activity was presented as percent of activity of the calB standard (Figs 2D and 3C). The specific calB activity was also calculated and presented in units per liter (U/L) or units per gram of total protein (U/g) (Supplementary Table 5), where the unit corresponds to the amount of enzyme which liberates 1 μmol pNP per minute with pNPB as a substrate. The total protein concentrations in the samples were analyzed by a standard Bradford method (BioRad Protein Assay).

### Secretion signal sequence screen

Several clones were randomly picked from each transformation plate to inoculate 4 mL of glucose medium cultivations in 24-well plates. Cells were cultivated for 4 days at 28 °C (800 rpm). Culture supernatant samples were diluted into water and analyzed by SDS-PAGE to assess production of the calB by the individual clones (Supplementary Fig. 1). In addition, the calB activity was analyzed in a subset of the samples (Supplementary Fig. 2). The results are presented as raw activity values without normalization to the total protein concentrations. For the both analyses, the CBHI-calB strain and the parental strain were used in the parallel cultivations as positive and negative controls, respectively.

### Gel-filtration chromatography and freeze-drying

The selected calB samples collected form bioreactor cultivations were subjected to gel-filtration chromatography using 50 mM potassium phosphate solution (pH 7.5) equilibrated Econo-Pac 10DG Desalting Columns (Bio-Rad). Four mL of supernatant samples collected on the last fermentation days (day 5 for the glucose-medium, or day 7 for the SG-lactose medium) were loaded onto the columns. Elution of the proteins was done with 3 mL of 50 mM potassium phosphate solution (pH 7.5). The eluent was divided into 1.5 mL tubes (one mL per tube) and the samples were frozen in liquid nitrogen and freeze-dried using Christ Alpha RVC instrument (0.05 mbar; B. Braun Biotech International). The freeze-dried samples were reconstituted by addition of one mL of water and the lipase activity was measured as described in the sections “calB activity test”.

## Supplementary information

Supplementary figures and tables
