# Supplementary material for: Novel genetic tools that enable highly pure protein production in Trichoderma reesei
Source: Sci Rep. 2019 Mar 22;9:5032. doi: 10.1038/s41598-019-41573-8 (PMC6430808; doi:10.1038/s41598-019-41573-8)
Supplement: Supplementary file 1 — Supplementary figures and tables [file 41598_2019_41573_MOESM1_ESM.pdf]

Novel genetic tools that enable highly pure protein production in *Trichoderma reesei*

Anssi Rantasalo, Marika Vitikainen, Toni Paasikallio, Jussi Jäntti, Christopher P. Landowski, and Dominik Mojzita*

VTT Technical Research Centre of Finland, P.O. Box 1000, FI-02044 VTT, Espoo, Finland

*Corresponding author: Tel: +358 40 3591913; Email: dominik.mojzita@vtt.fi

Supplementary figures

Supplementary figure 1: Screen for efficient secretion signal sequences - SDS-PAGE analysis

Supplementary figure 2: Screen for efficient secretion signal sequences - calB enzymatic activity

Supplementary figure 3: Construction and analysis of additional AaSS-calB strains

Supplementary figure 4: Transcription levels of selected genes encoding proteins involved in secretion

Supplementary figure 5: Original scans of the SDS-PAGE gels shown in Figures 2C and 3B

Supplementary tables

Supplementary table 1: Secretion signal sequences tested in this study

Supplementary table 2: Primers used for construction of cellulose-loci targeted cassettes (SES constructs)

Supplementary table 3: Primers used for the gene copy number and deletion analyses

Supplementary table 4: Primers used for the transcription analysis

Supplementary table 5: The specific activities of calB produced by the SES strains in bioreactors

Supplementary Figure 1

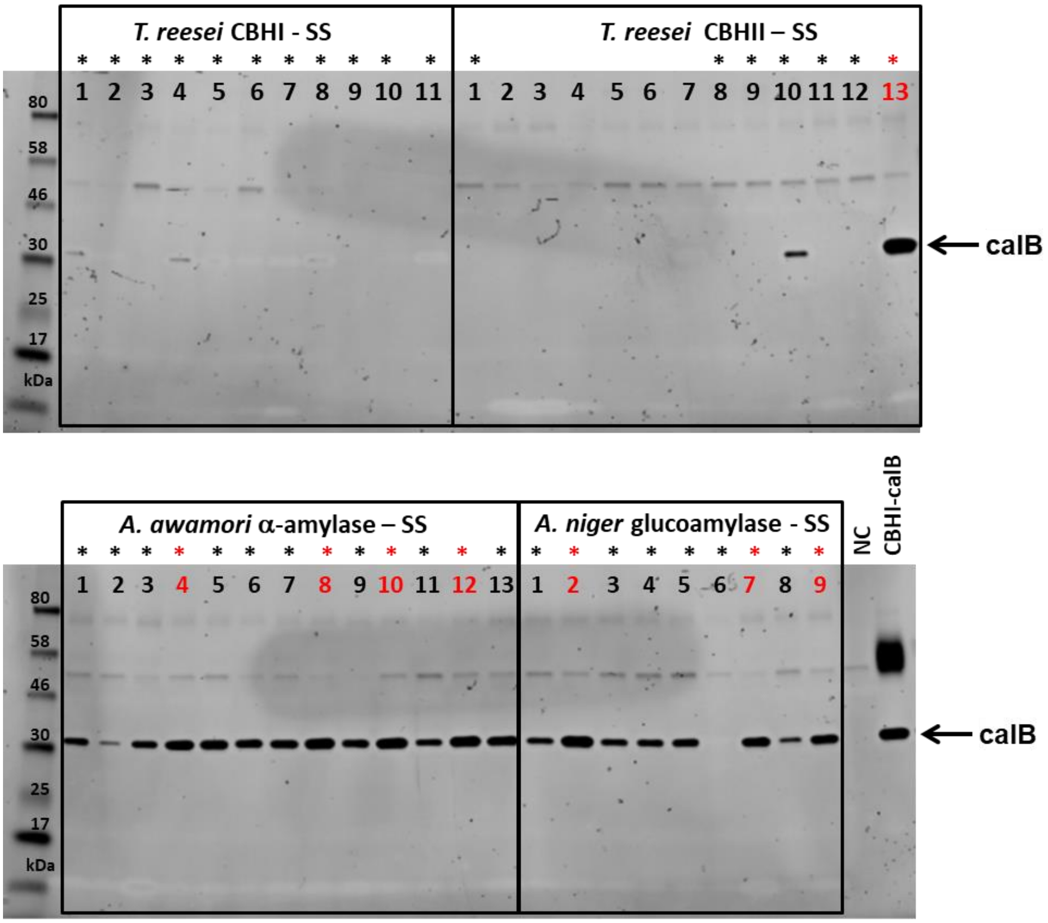

Supplementary figure 1: Screen for efficient secretion signal sequences - SDS-PAGE analysis

The secretion signal sequences (SS) of CBHI (*T. reesei*), CBHII (*T. reesei*), α-amylase (*A. awamori*) and glucoamylase (*A. niger*) (see **Supplementary table 2**) were fused to the calB encoding sequence to form the N-terminus of resulting proteins. The sTF and SS-calB expression cassettes were integrated into T. reesei genome by two steps of CRISPR/Cas9 mediated transformations. In the first step, the sTF expression cassette was targeted into the *egl1* locus. And in the second step, the resulting strain was transformed by multiplexed CRISPR/Cas9 targeting the SS-calB cassettes simultaneously into the *cbh1*, *cbh2*, and *egl2* loci (as described in the main text and shown in **Figure 3A**). Several colonies (9-13) were randomly selected from each transformation plate and the strains cultivated in 4 mL of glucose-containing medium in 24-well plate for 3 days. The total protein produced (secreted) was analyzed by the SDS-PAGE (Coomassie staining) to test the efficiency of each signal sequence for the calB protein secretion. The culture media samples were diluted 1:10 in water and 12 μL were loaded into the gel. The previously tested CBHI-calB strain (SES strain, Figure 2A) and its parental strain, lacking the sTF and the CBHI-calB expression cassettes, were included in the parallel cultivations as positive and negative control (NC), respectively. The culture supernatant samples collected from clones marked with asterisk (*) were further analyzed by performing the lipase assay. The clones highlighted in red were also subjected to gene copy number analysis (data not shown), from which the only correct clone was found to be clone #4 of the calB-producer with α-amylase-SS, containing three copies of the SS-calB cassettes and all three corresponding cellulase genes deletions.

Supplementary Figure 2

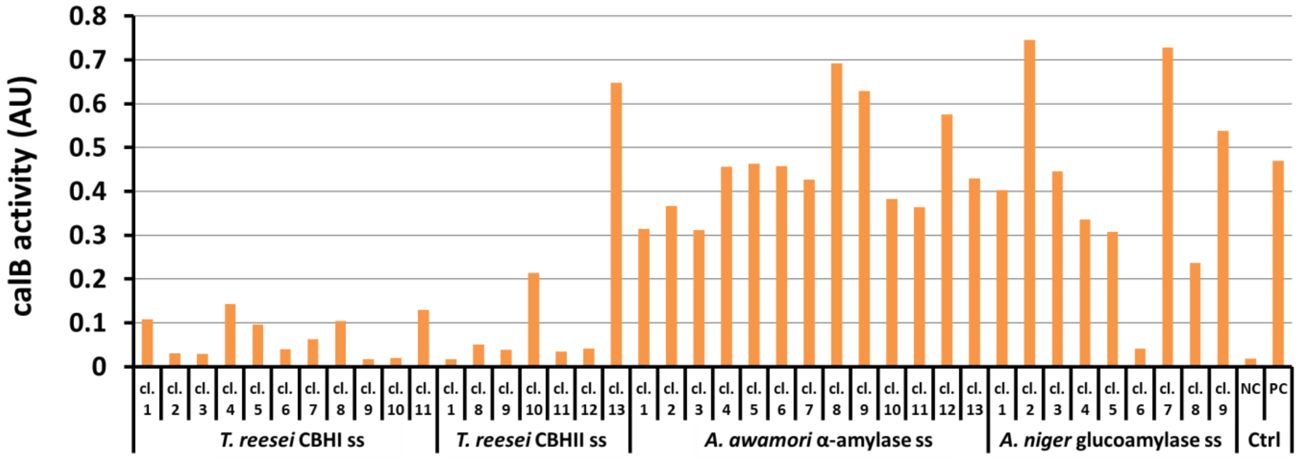

Supplementary figure 2: Screen for efficient secretion signal sequences - calB enzymatic activity

The selected samples described in Supplementary figure 1 were analyzed for the calB-lipase activity. The lipase activity levels shown here represent raw measurement values from a single technical replicate (no normalization to protein concentration). The culture supernatants from the CBHI-calB strain (SES strain, **Figure 2A**) and its parental strain, lacking the sTF and the CBHI-calB expression cassettes, were included in the calB activity measurements as positive (PC) and negative controls (NC), respectively.

Supplementary Figure 3

A

	Triple deletion	Double deletion			Single deletion			No deletions	
	Δ*cbh1* Δ*cbh2* Δ*egl2*	Δ*cbh1* Δ*cbh2*	Δ*chb1* Δ*egl2*	Δ*cbh2* Δ*egl2*	Δ*cbh1*	Δ*cbh2*	Δ*egl2*		Total
Number of clones	**9 (15%)**	**1 (2%)**	**0 (0%)**	**32 (53%)**	**1 (2%)**	**5 (8%)**	**4 (7%)**	**8 (13%)**	**60**
Pure clones (% of total)	**5 (56%)**	**0 (0%)**	**0 (0%)**	**27 (84%)**	**0 (0%)**	**5 (100%)**	**3 (75%)**	**-**	**40 (67%)**
Mixed clones (% of total)	**4 (44%)**	**1 (100%)**	**0 (0%)**	**5 (16%)**	**1 (100%)**	**0 (0%)**	**1 (25%)**	**-**	**12 (20%)**

B

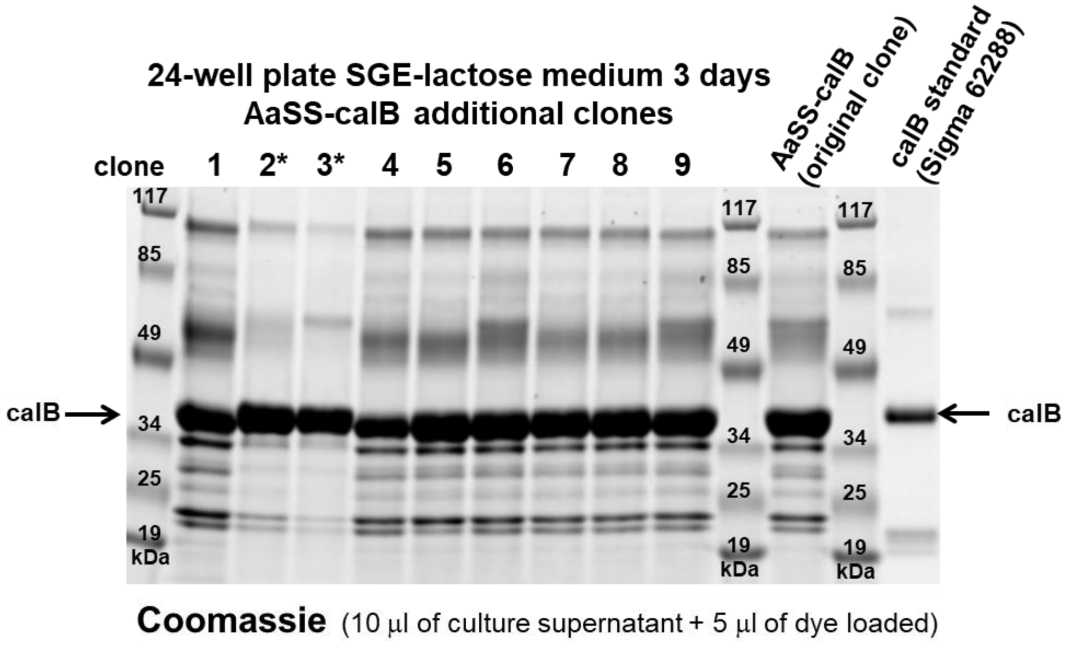

Supplementary figure 3: Construction and analysis of additional AaSS-calB strains

The AaSS-calB strain was constructed again by multiplexed CRISPR/Cas9 targeting the AaSS-calB cassettes simultaneously into the *cbh1*, *cbh2*, and *egl2* loci (as described in the main text and shown in Figure 3A). **A)** Sixty colonies were randomly selected from the transformation plate and analyzed for correct replacements of the targeted loci by the AaSS-calB cassette. Nine out of 60 colonies contained all three target genes deleted; 5 pure clones were obtained directly, and additional 4 clones were obtained by purification of mixed colonies by plating diluted conidia and re-analyzing the resulting colonies. **B)** The additional AaSS-calB colonies were cultivated next to the originally isolated AaSS-calB strain in 4 mL of SG-lactose medium in 24-well plate for 3 days. The SDS-PAGE analysis of the cultures supernatants revealed highly similar amounts of calB produced in the tested strains. The clones 2 and 3 (marked with asterisk) contained more than three copies of the AaSS-calB cassette integrated in the genome as determined by qPCR.

Supplementary Figure 4

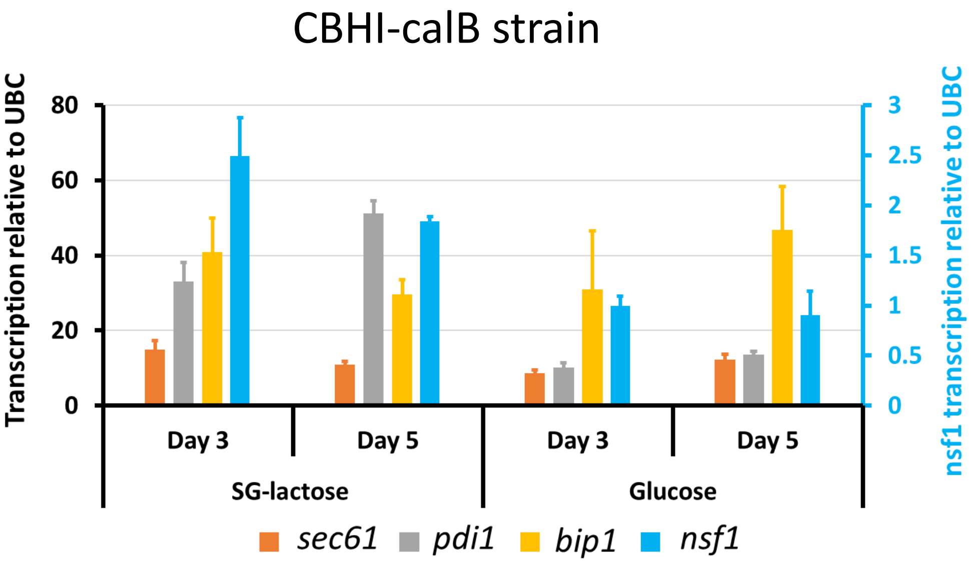

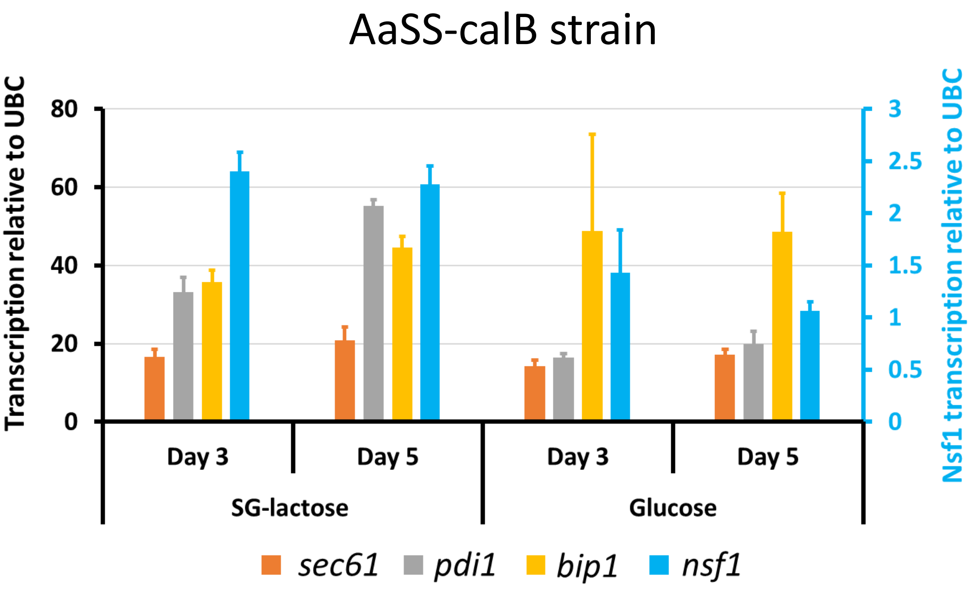

Supplementary figure 4: Transcription levels of selected genes encoding proteins involved in secretion

The effect of cultivation conditions on transcriptional regulation of selected genes involved in protein secretion was studied. The analysis was performed on CBHI-calB (**Figure 2A**) and AaSS-calB (**Figure 3A**) strains grown in bioreactors in cellulase inducing SG-lactose and cellulase repressing glucose media. The analysis was performed on samples collected on the days 3 and 5. Transcription levels of selected genes were normalized to the transcript levels of endogenous *ubc* gene. Transcript levels of the *nsf1* are shown on the secondary y-axis (blue axis on the right). Values and error bars represent the mean and standard deviation from two biological (four technical) replicates.

Supplementary Figure 5

A

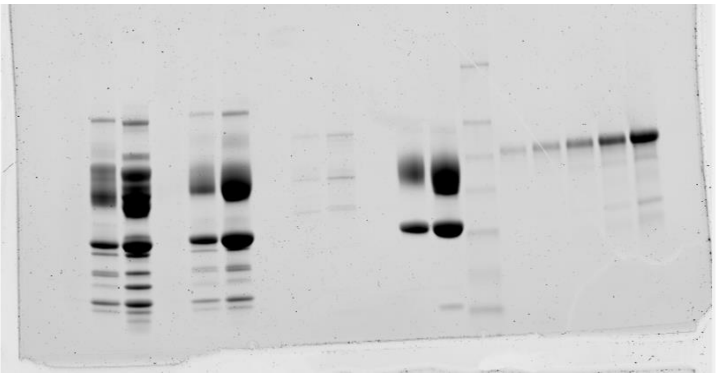

B

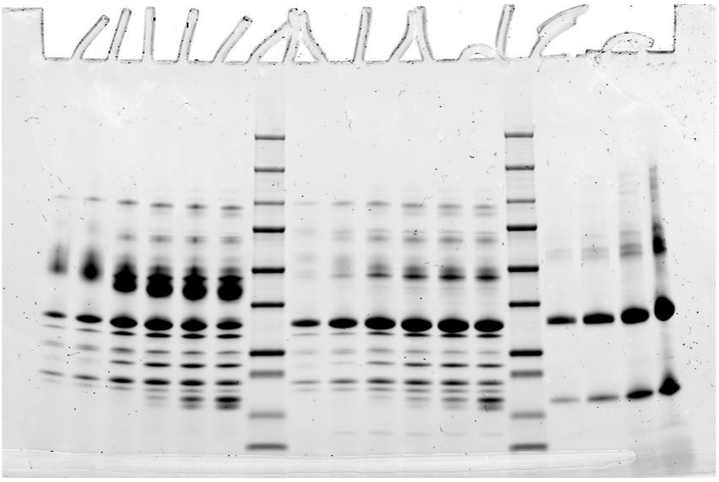

C

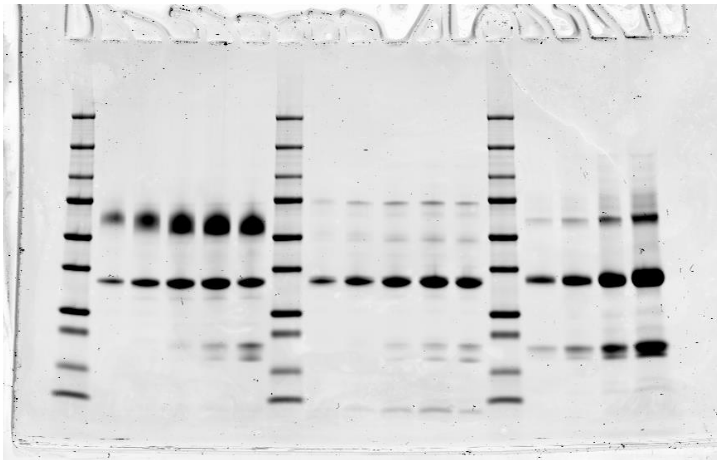

Supplementary figure 5: Original scans of the SDS-PAGE gels shown in Figures 2C and 3B.

The un-processed images of Coomassie-stained gels as acquired by the Odyssey CLx Imaging System instrument (LI-COR Biosciences). **A)** The original gel scan shown here was rotated, cropped, and the values of molecular weight standards were added before the use in **Figure 2C**. **B)** The original gel scan shown here was rotated, cropped, and the values of molecular weight standards were added before the use in **Figure 3B** (left). **C)** The original gel scan shown here was cropped, and the values of molecular weight standards were added before the use in **Figure 3B** (right).

Supplementary Table 1

Source gene	Organism	Amino acid sequence	Reference
α-amylase	*Aspergillus awamori*	MRVSTSSLALSVSLFGKLALGLSAAE	39
Glucoamylase	*Aspergillus niger*	MSFRSLLALSGLVCTGLANV	39
cbh1	*Trichoderma reesei*	MYRKLAVISAFLATARAQSA	38
cbh2	*Trichoderma reesei*	MIVGILTTLATLATLAASVPL	39

Supplementary Table 1: Secretion signal sequences tested in this study. The first N-terminal 23 amino acids of the calB were replaced by the signal sequence. The references are numbered based on the main text.

Supplementary Table 2

Target	Location relative to CDS	Primer name	Sequence
cbh1 - 5'	1581 to 2579 bp upstream of start codon	CBH1_5_GA_F	CTATAGGGAGACCACAACGGTTTCCCTGTTTAAACGCTAGTTTGCGCTCACACCAC
		CBH1_5_GA_R	GAGCTTGTACAGCTAGCGGCCGCAAAATACACAGAAGACACAACAAGTATCCAGTTCTTC
cbh1 - 3'	0 to 1000 bp downstream of stop codon	CBH1_3_GA_F	TTGCGGCCGCTAGCTGTACAAGCTCCGTGGCGAAAGCCTG
		CBH1_3_GA_R	CGGGCTTTGTTAGCAGCCGGATCAGTTTAAACGGATTTTGGAGGCAGCACATTC
cbh2 - 5'	1554 to 2553 bp upstream of start codon	CBH2_5_GA_F	CTATAGGGAGACCACAACGGTTTCCCTGTTTAAACATACTCCGGCCGGCTGTGCA
		CBH2_5_GA_R	AGCCTGTACAGCTAGCGGCCGCTTAGGGCAATGACGTAGTAGGTATATGC
cbh2 - 3'	0 to 1000 bp downstream of stop codon	CBH2_3_GA_F	AAGCGGCCGCTAGCTGTACAGGCTTTCGTGACCGGGCTTC
		CBH2_3_GA_R	CGGGCTTTGTTAGCAGCCGGATCAGTTTAAACGGTTATCCACGACAGCCATATTC
egl1 - 5'	811 to 1811 bp upstream of start codon	EGL1_5_GA_F	CTATAGGGAGACCACAACGGTTTCCCTGTTTAAACGTATCCCAGCTTGCGCATCC
		EGL1_5_GA_R	GCTTGTACAGCTAGCGGCCGCTTCGAAGTCGATCAGACTGTTTG
egl1 - 3'	2 to 1001 bp downstream of stop codon	EGL1_3_GA_F	CGAAGCGGCCGCTAGCTGTACAAGCGTTGACTTGCCTCTGGTC
		EGL1_3_GA_R	CGGGCTTTGTTAGCAGCCGGATCAGTTTAAACGTTCAAGGGCCTCCTCTACT
egl2 - 5'	1248 to 2247 bp upstream of start codon	EGL2_5_GA_F	CTATAGGGAGACCACAACGGTTTCCCTGTTTAAACTTGGTAAGTCTTTCTTCCGCTTGC
		EGL2_5_GA_R	AGTGTGTACAGCTAGCGGCCGCGTTGGTTCGTGTGCAAGGTTG
egl2 - 3'	1 to 1000 bp downstream of stop codon	EGL2_3_GA_F	AACGCGGCCGCTAGCTGTACACACTCTGAGCTGAATGCAGAAG
		EGL2_3_GA_R	CGGGCTTTGTTAGCAGCCGGATCAGTTTAAACGGTTCGACAAGTCCAAAGGTAG

Supplementary Table 2: Primers used for construction of cellulose-loci targeted cassettes (SES constructs). The PCR products obtained from the *T. reesei* genomic DNA were used as DNA regions flanking the SES-expression cassettes in the donor DNA molecules used for genomic integrations/ replacements of the selected genes. The *T. reesei* genome database ID numbers for the selected loci are listed in Supplementary Table 3.

Supplementary Table 3

Target	JGI database ID *	Primer name	Sequence
act1	44504	act1_qPCR_gDNA_F	GCCTTCTATGTCTCCATCCAG
		act1_qPCR_gDNA_R	CTCAGCCAGGATCTTCATCAG
Bm3R1		BM3R1_qPRC_F	GTCACATTCACAAAGAACCATCC
		BM3R1_qPRC_R	GACCTCCATAAACGATCCGA
calB		calB_qPCR_F	ACGTTCTTTCCCAGCATCC
		calB_qPCR_R	GCTGTAGAGGTTGGTGGTG
xyn1	74223	g-xyn1_qPCR_F	CTGCTTTCCGTCTATGGCTG
		g-xyn1_qPCR_R	GTACTGGTTGAAGGTCGCTG
cbh1	123989	g-cbh1_qPCR_F	ACCACCAAGAAATTGACCGT
		g-cbh1_qPCR_R	CGCCCTTGTCTGAGAAAGAG
cbh1 promoter		cbh1pr_qPCR_F	GTTTGTTTCTTCACTCAGTCCA
		cbh1pr_qPCR_R	CGATACACTCAAGCATCCCT
cbh2	72567	g-cbh2_qPCR_F	CATCAACTACGCCGTCAC
		g-cbh2_qPCR_R	GCTGGTAATGTTCCACCC
egl1	122081	g-egl1_qPCR_F	ACCTTCACCATCATCACCC
		g-egl1_qPCR_R	TCCAAATGCTGAACACGAG
egl2	120312	g-egl2_qPCR_F	CTTGATTCCACGAGCATTTCC
		g-egl2_qPCR_R	CATGATGCCGAACCACAC

Supplementary Table 3: Primers used for the gene copy number and deletion analyses by RT-PCR. * - corresponding protein ID in the Joint Genome Institute's database (http://genome.jgi.doe.gov/programs/fungi/index.jsf).

Supplementary Table 4

Target	JGI database ID*	Primer name	Sequence
ubc	77732	UBC6_qPCR_F	CCAACATCCTCGAATGGCAC
		UBC6_qPCR_R	GCTGGGTTGAAGGATTTCGG
Bm3R1		BM3R1_qPRC_F	GTCACATTCACAAAGAACCATCC
		BM3R1_qPRC_R	GACCTCCATAAACGATCCGA
calB		calB_qPCR_F	ACGTTCTTTCCCAGCATCC
		calB_qPCR_R	GCTGTAGAGGTTGGTGGTG
sec61	121397	sec61_qPCR_F	GCTGTCACCATCATCTACGG
		sec61_qPCR_R	GGCTAGAATTTCCATGCAAGTC
pdi1	122415	pdi1_qPCR_F	CAAGGAGATCACCGAGAAGGA
		pdi1_qPCR_R	AGCGTAGAACTCAATCAGGAC
bip1	122920	bip1_qPCR_F	CCATCACCATCACCAACGAC
		bip1_qPCR_R	GTCAAGAATAGTCTCCTTGTCCT
nsf1	62040	nsf1_qPCR_F	GGAGATGATGCGTGTTGTGG
		nsf1_qPCR_R	GTAGTCCTTCTTGGCGAACTC

Supplementary Table 4: Primers used for the transcription analysis performed by RT-PCR. * - corresponding protein ID in the Joint Genome Institute's database (http://genome.jgi.doe.gov/programs/fungi/index.jsf).

Supplementary Table 5

			U/L	U/g
SG+lactose	CBHI-calB	Day 1	**44 ± 3**	**69 ± 4**
		Day 2	**55 ± 3**	**82 ± 4**
		Day 3	**912 ± 38**	**218 ± 9**
		Day 4	**2753 ± 78**	**341 ± 10**
		Day 5	**5278 ± 75**	**303 ± 4**
		Day 6	**6076 ± 109**	**284 ± 5**
		Day 7	**6457 ± 202**	**302 ± 9**
		Day 7*	**6560 ± 209**	**347 ± 11**
	AaSS-calB	Day 1	**46 ± 3**	**80 ± 6**
		Day 2	**72 ± 1**	**113 ± 1**
		Day 3	**2082 ± 113**	**497 ± 27**
		Day 4	**5006 ± 140**	**697 ± 19**
		Day 5	**7137 ± 28**	**566 ± 2**
		Day 6	**8171 ± 83**	**538 ± 5**
		Day 7	**8810 ± 221**	**505 ± 13**
		Day 7*	**8687 ± 231**	**578 ± 15**
Glucose	CBHI-calB	Day 2	**626 ± 42**	**289 ± 19**
		Day 3	**1998 ± 150**	**366 ± 27**
		Day 4	**3567 ± 110**	**477 ± 15**
		Day 5	**5312 ± 150**	**531 ± 15**
		Day 5 *	**5305 ± 172**	**574 ± 19**
	AaSS-calB	Day 2	**935 ± 71**	**904 ± 69**
		Day 3	**2335 ± 131**	**817 ± 46**
		Day 4	**4184 ± 184**	**1132 ± 50**
		Day 5	**5820 ± 177**	**1263 ± 38**
		Day 5*	**5372 ± 173**	**1189 ± 38**
calB standard (Sigma 62288)			n/a	**793 ± 5**

Supplementary Table 5: The specific activities of calB produced by the SES strains in bioreactors. The CBHI-calB and the AaSS-calB strain were cultivated either in SG-lactose medium for 7 days or in glucose-containing medium for 5 days. The calB activity was measured in the cultivation media samples (same as in **Figure 3C**), and are presented in units per liter of culture supernatant (U/L) or units per gram of total protein (U/g). The unit corresponds to the amount of enzyme which liberates 1 μmol pNP per minute with pNPB as a substrate. The samples from the last day of each cultivation subjected to gel-filtration chromatography and freeze-drying were analyzed after reconstitution in water (marked with asterisk). The values and the error bars represent means and standard deviations from three technical replicates.
